# Identification and Validation of a Potent Dual Inhibitor of the *P*. *falciparum* M1 and M17 Aminopeptidases Using Virtual Screening

**DOI:** 10.1371/journal.pone.0138957

**Published:** 2015-09-25

**Authors:** Chiara Ruggeri, Nyssa Drinkwater, Komagal Kannan Sivaraman, Rebecca S. Bamert, Sheena McGowan, Alessandro Paiardini

**Affiliations:** 1 Dipartimento di Scienze biochimiche "A. Rossi Fanelli", Sapienza Università di Roma, Piazzale Aldo Moro 5, 00185, Roma, Italy; 2 Department of Microbiology, Monash University, Clayton Campus, Melbourne, Victoria, 3800, Australia; 3 Department of Biochemistry and Molecular Biology, Monash University, Clayton Campus, Melbourne, Victoria, 3800, Australia; 4 Dipartimento di Biologia e Biotecnologie "Charles Darwin", Sapienza Università di Roma, Piazzale Aldo Moro 5, 00185, Roma, Italy; Tulane University, UNITED STATES

## Abstract

The *Plasmodium falciparum Pf*A-M1 and *Pf*A-M17 metalloaminopeptidases are validated drug targets for the discovery of antimalarial agents. In order to identify dual inhibitors of both proteins, we developed a hierarchical virtual screening approach, followed by *in vitro* evaluation of the highest scoring hits. Starting from the ZINC database of purchasable compounds, sequential 3D-pharmacophore and molecular docking steps were applied to filter the virtual ‘hits’. At the end of virtual screening, 12 compounds were chosen and tested against the *in vitro* aminopeptidase activity of both *Pf*A-M1 and *Pf*A-M17. Two molecules showed significant inhibitory activity (low micromolar/nanomolar range) against both proteins. Finally, the crystal structure of the most potent compound in complex with both *PfA-*M1 and *PfA-*M17 was solved, revealing the binding mode and validating our computational approach.

## Introduction

According to recent statistics from the World Health Organization (WHO), malaria affects more than 225 million individuals, causing approximately 600 000 deaths each year [1]. The parasite *P*. *falciparum* is the most lethal of the *Plasmodium* species that cause human disease [2]. Clinical disease symptoms, including fever, headache, anemia, respiratory distress and blockage of deep capillaries, are caused by a repeated cycle of erythrocyte invasion and lysis by asexual blood stage parasites. The majority of current therapies target this stage of the parasite life-cycle [3]. The lack of an effective vaccine and emerging resistance to front-line antimalarials, including the artemisinins, poses a global public health threat and demands the development of next generation antimalarial agents [4].

One pathway that has attracted the attention of antimalarial drug discovery efforts is the catabolism of erythrocyte hemoglobin, which is catalyzed by several enzymes and therefore presents a number of potential therapeutic targets [3]. Among these novel targets are the aminopeptidase enzymes that remove N-terminal amino acids from short peptides with high specificity. The *P*. *falciparum* alanyl aminopeptidase, *Pf*A-M1, and leucyl aminopeptidase, *Pf*A-M17, act in concert to mediate the final stages of hemoglobin digestion [5,6]. *Pf*A-M1 has broad substrate specificity, preferentially cleaving P1 residues Leu, Ala, Arg and Lys; however, it can also cleave Phe, Tyr, Asn and Ser [7]. In contrast, *Pf*A-M17 demonstrates a restricted specificity for Leu and, to a lesser extent, Ala [8, 9]. The active sites of *Pf*A-M1 and *Pf*A-M17 coordinate essential zinc ions that are required for the catalytic mechanism. *Pf*A-M1 coordinates a single zinc metal ion, while *Pf*A-M17 contains two metal binding sites [10]. The two aminopeptidases are each encoded by non-homologous genes and have been validated *in vitro* and *in vivo* as drug targets, as inhibition of their activity can control both murine and laboratory malaria parasites [10]. Previous work within our group has identified potent dual inhibitors of the enzymes [7, 9, 11–14], which bind via coordination of the zinc ions by a zinc binding group (ZBG).

Virtual screening is now established as a valuable tool in early drug discovery, allowing fast and economical selection of “hit” molecules before, subsequent experimental validation of the virtual hits. This biological validation is absolutely required; indeed, in recent years several virtual screening campaigns have been undertaken, with many papers reporting “hits” from virtual screens that haven’t been evaluated experimentally [15,16]. Virtual screening can add significant value to a drug discovery campaign; however, it demands careful attention to methodology with regard to design, validation and experimental confirmation of the computational results.

We were interested to evaluate whether a virtual screening study could identify novel molecules that are capable of dual inhibitors of both *Pf*A-M1 and *Pf*A-M17. To this end, we undertook a virtual screen of the ZINC database of purchasable subsets (~18 millions of compounds) [17,18] and used successive 3D-pharmacophore and molecular docking to filter the virtual ‘hits’. Our screen identified 12 compounds that satisfied both the 3D-pharmacophore and docking requirements. We investigated the inhibitory properties of the 12 compounds against the aminopeptidase activity of both *Pf*A-M1 and *Pf*A-M17 *in vitro*, and demonstrated that that two compounds were dual *Pf*A-M1/*Pf*A-M17 inhibitors. Finally, we determined the crystal structures of the most potent hit in complex with both *Pf*A-M1 and *PfA-*M17. Despite some discrepancy between the predicted and experimentally determined poses of the most potent hit, the obtained results demonstrate that, overall, the presented virtual screening protocol was able to effectively identify dual inhibitors for *Pf*A-M1 and *Pf*A-M17.

## Materials and Methods

### Structure-based virtual screening

#### Generation of a structure-based pharmacophore model

The structure-based pharmacophore model was generated using LigandScout software package v.3.0 [19]. The models were generated from PDB codes 3EBH and 3EBI for *Pf*A-M1, and 3KR4 and 3KR5 for *Pf*A-M17 [6–9]. We superposed the two inhibitors bestatin and hPheP[CH_2_]Phe for both drug targets and generated a shared-features 3D pharmacophore. The pharmacophore model was validated by screening with a manually generated database of compounds having bestatin and hPheP[CH_2_]Phe seeded into 100 decoys from DUD-E decoy compounds database [20]. During the initial validation step, the first generated pharmacophore model was associated with poor performance. Therefore, pharmacophore maps were manually modified by systematically including/removing features, by increasing/reducing features tolerance and by adjusting the exclusion volume spheres, in order to extract only bestatin and hPheP[CH_2_]Phe from the database of decoys.

#### Virtual screening

Virtual screening was carried out using ZincPharmer [18] and LigandScout software package v. 3.1 [19], using the previously obtained pharmacophore models, to search the Zinc database of fixed conformers [17]. A maximum of 0.5 Å Root Mean Square Deviation (RMSD) from sphere centers, 10 rotatable bonds cut-off and molecular weight in the range of 180–500 Daltons were used as input parameters for ZincPharmer. Compounds were considered potential hits and retrieved for further analysis if they satisfied at least (*n*/2) + 1 features of the pharmacophore models.

#### Docking settings

The compounds retrieved from the pharmacophore search were used as input for a docking study. Molegro Virtual Docker ver. 5.5 (MVD) [21] and FlexX [22] were used for a more accurate prediction, compared to pharmacophore search, of the binding mode inside the active site of *Pf*A-M1 and *Pf*A-M17. An initial re-docking of bestatin and hPheP[CH_2_]Phe into the active sites of the two aminopeptidases was carried out with MVD, in order to define the correct parameters to be used during the simulations. The final selected values for grid center were: X = 75.94 Y = 65.02 Z = 76.45 for *Pf*A-M1 and X = 88.03 Y = 74.43 Z = 29.88 for *Pf*A-M17, respectively. The grid sphere radius was set to 12.0 Å in both systems. The “Moldock” [21] optimizer algorithm was chosen as a search algorithm using the following values: 10 numbers of run; 50 number of individuals in the population; water molecules were excluded from the docking simulations. A second docking simulation was carried out with FlexX, a tool implemented in the Lead-It software package (®Biosolve IT) that is specifically suited for predicting coordination geometries in zinc containing metalloproteins. The active site was defined including all residues within a 10.0 Å radius sphere from the center of the mass of the ligand. The clash factor was set to 0.6. Others parameter were kept as default [22].

#### Post-filtering of docking results

The virtual molecule hits obtained from the docking-based virtual screening step were re-ranked according to their RMSD, considering only those compounds with a similar pose in both docking tools (RMSD < 2.0 Å). The top-ranked poses were re-assessed using the “Hyde” scoring function implemented in the Lead-It software package [23]. 12 hits were chosen on the basis of the estimated free-energy of binding and purchase availability.

### Biochemistry

#### Expression, purification and enzyme assays

The expression and purification of *Pf*A-M1 and *Pf*A-M17 in *Escherichia coli* employed a two-step purification process of Ni-NTA-agarose column, followed by size exclusion chromatography on a Superdex 200 16/60 using an AKTAxpress high throughput chromatography system (http://proteinexpress.med.monash.edu.au/index.htm), as previously described [12,13]. Compounds were purchased from Ambinter (France). Purity (90% or higher) of these compounds was confirmed by vendors. Aminopeptidase activity and *K*_i_ values for both enzymes were determined as already described [14,15].

#### Crystallization, data collection, structure solution and refinement

Crystals of the *Pf*A-M1 and *Pf*A-M17 in complex with compound 12 were obtained by soaking as described previously [12, 13]. Briefly, *Pf*A-M1 was concentrated to 8.0 mg/mL in 50 mM Hepes pH 8.0, 300 mM NaCl and 5% glycerol. Unliganded crystals were grown by hanging-drop vapour diffusion, in 20% PEG8000, 0.2M MgCl2, 0.1M Tris pH 8.5, and 10% glycerol. The crystallisation solution was supplemented with 2 mM of compound 12 to make the soak solution. Crystals were soaked in compound soak solution overnight prior to data collection. *Pf*A-M17 was concentrated to 13 mg/mL in 50 mM Hepes pH 8.0, 300 mM NaCl. Unliganded crystals were grown in 40% PEG400, 0.1M Tris pH 8.4, and 0.2M LiSO4. The crystallisation solution was supplemented with 2 mM of compound 12 and 1 mM ZnSO4 to make the soak solution, in which they were soaked for 2 days prior to data collection. Data were collected at 100 K using synchrotron radiation at the Australian Synchrotron using the macro-crystallography MX1 beamline 3BM1 for *Pf*A-M1 [24], and the micro-crystallography beamline for *Pf*A-M17. Diffraction images were processed and integrated using iMosflm [25] (*Pf*A-M1) or XDS [26] (*Pf*A-M17) and scaled using aimless from the CCP4 suite [27]. The model was refined in Phenix [28] and manually built into electron density using Coot [29]. Summary statistics are provided in Table 1. The structures were deposited in the protein databank with accession codes 4ZQT and 5CBM.

**Table 1:** Data collection and refinement statistics.

	*Pf*A-M1–12	*Pf*A-M17–12
PDB ID	4ZQT	5CBM
***Data collection***		
Resolution range (Å)	62–1.98 (2.05–1.98)	49–2.30 (2.33–2.30)
Space group	*P*2_1_2_1_2_1_	*P*2_1_2_1_2_1_
Unit cell	a = 75.4; b = 109.1; c = 118.1 α = β = γ = 90°	a = 174.1; b = 177.7; c = 231.0 α = β = γ = 90°
Total reflections	762957 (68374)	2509800 (122968)
Unique reflections	68268 (6667)	315610 (15462)
Multiplicity	11.2 (10.3)	8.0 (8.0)
Completeness (%)	99.9 (99.3)	99.9 (99.7)
Mean I/sigma(I)	20.0 (2.5)	4.9 (1.1)
R-merge	0.678 (1.90)	0.482 (3.64)
R-meas	0.711	0.516
CC1/2	0.853 (0.388)	0.982 (0.446)
***Refinement statistics***		
R-work	0.1626	0.1821
R-free	0.2080	0.2346
# of non-hydrogen atoms	8179	50850
macromolecules	7241	46963
ligands	35	194
solvent	903	3693
RMS(bonds)	0.008	0.008
RMS (angles)	0.99	1.09
Ramachandran favored (%)	98	97
Ramachandran outliers (%)	0.11	0.24
Clashscore	4.17	3.39
Average B-factor	23.60	26.91
macromolecules	22.40	26.60
ligands	27.10 (28*)	20.15 (24*)
solvent	33.60	31.82
Molprobity Score	1.2; 100^th^ percentile (N = 12290, 1.98 Å ± 0.25Å)	1.4; 99^th^ percentile (N = 8909, 2.30 Å ± 0.25Å)

* After an additional round of refinement including TLS parameters.

## Results

### Generation of 3D-pharmacophore hypotheses

**Fig 1:**
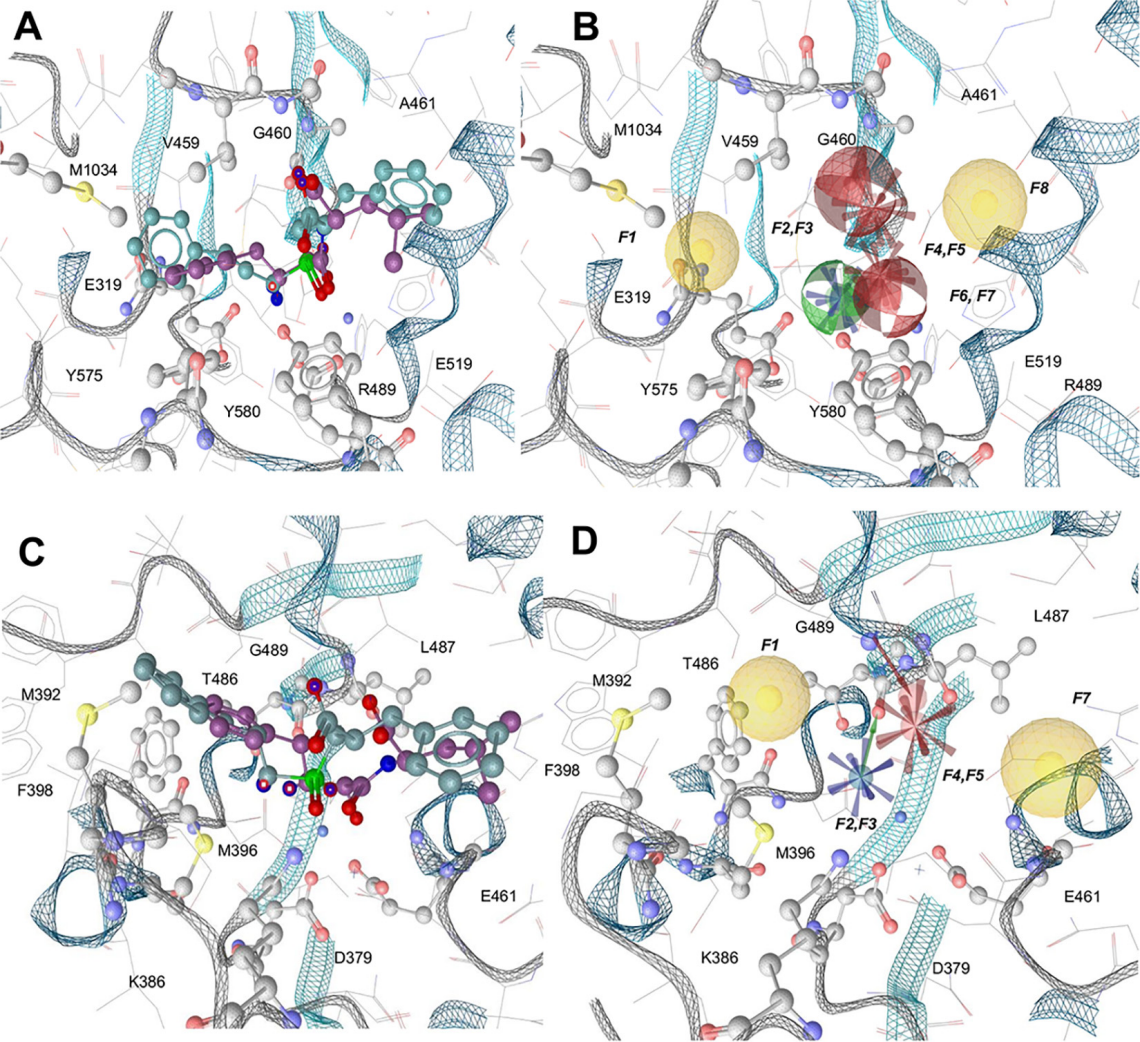
Pharmacophore hypotheses for *Pf*A-M1 and *Pf*A-M17. Protein backbone is shown as grey (loops) and cyan (strands and helices) ribbons. The residues interacting with the pharmacophore maps are represented as ball and sticks, and colored by atom type. Bestatin is represented in violet, while hPheP[CH_2_]Phe is in cyan. A) Superposition of bestatin and hPheP[CH_2_]Phe in the *Pf*A-M1 active site. B) *Pf*A-M1 obtained shared pharmacophore map, which consists of the following features: F1 and F8 hydrophobic features, F2 hydrogen bond donor feature, F3 positively charged feature, F4 and F7 hydrogen bond acceptor features, F5 and F6 negatively charged features. C) Superposition of bestatin and hPheP[CH_2_]Phe in the *Pf*A-M17 active site. D) *Pf*A-M17 obtained shared pharmacophore map, which consists of the following features: F1 and F6 hydrophobic features, F2 positively charged feature, F3 hydrogen bond donor feature, F4 negatively charged feature, F5 hydrogen bond acceptor feature.

With the aim of pinpointing the necessary key features of a potential dual *Pf*A-M1 and *Pf*A-M17 inhibitor, a pharmacophore hypothesis was generated for each target. We started from two well-characterized dual inhibitors, bestatin (2-(3-amino-2-hydroxy-4-phenyl-butyrylamino)-4-methyl-pentanoic acid) and hPheP[CH_2_]Phe, a phosphinate dipeptide analogue, for which high-resolution structures were available [7, 9], to derive a 3D-pharmacophore map representing the main interactions between the enzymes and inhibitors (Fig 1).

For *Pf*A-M1, the resulting map showed a total of eight pharmacophore features (Fig 1A & 1B). These included two hydrophobic patches (pointing towards the hydrophobic clefts formed by residues Met1034, Tyr575, Val459, Ala461 and Glu519) and three charged patches (one positively charged, deriving from the potential interaction with residues Glu319 and Glu519, and two negatively charged, arising from the interaction with the zinc ion and Arg489). Hydrogen bonding donors and acceptors were also observed at Ala461 and Gly460 (acceptors) and Tyr580 (donor). For *Pf*A-M17 (Fig 1C & 1D) six points of interest were included in the pharmacophore map: two hydrophobic points (formed by Met369, Phe398, Met392); a negatively charged feature (pointing to Lys386); a positively charged feature (near Asp379 and Glu461); a hydrogen bond donor (pointing to Thr486); a hydrogen bond acceptor (interacting with the main-chain of Gly489).

**Fig 2:**
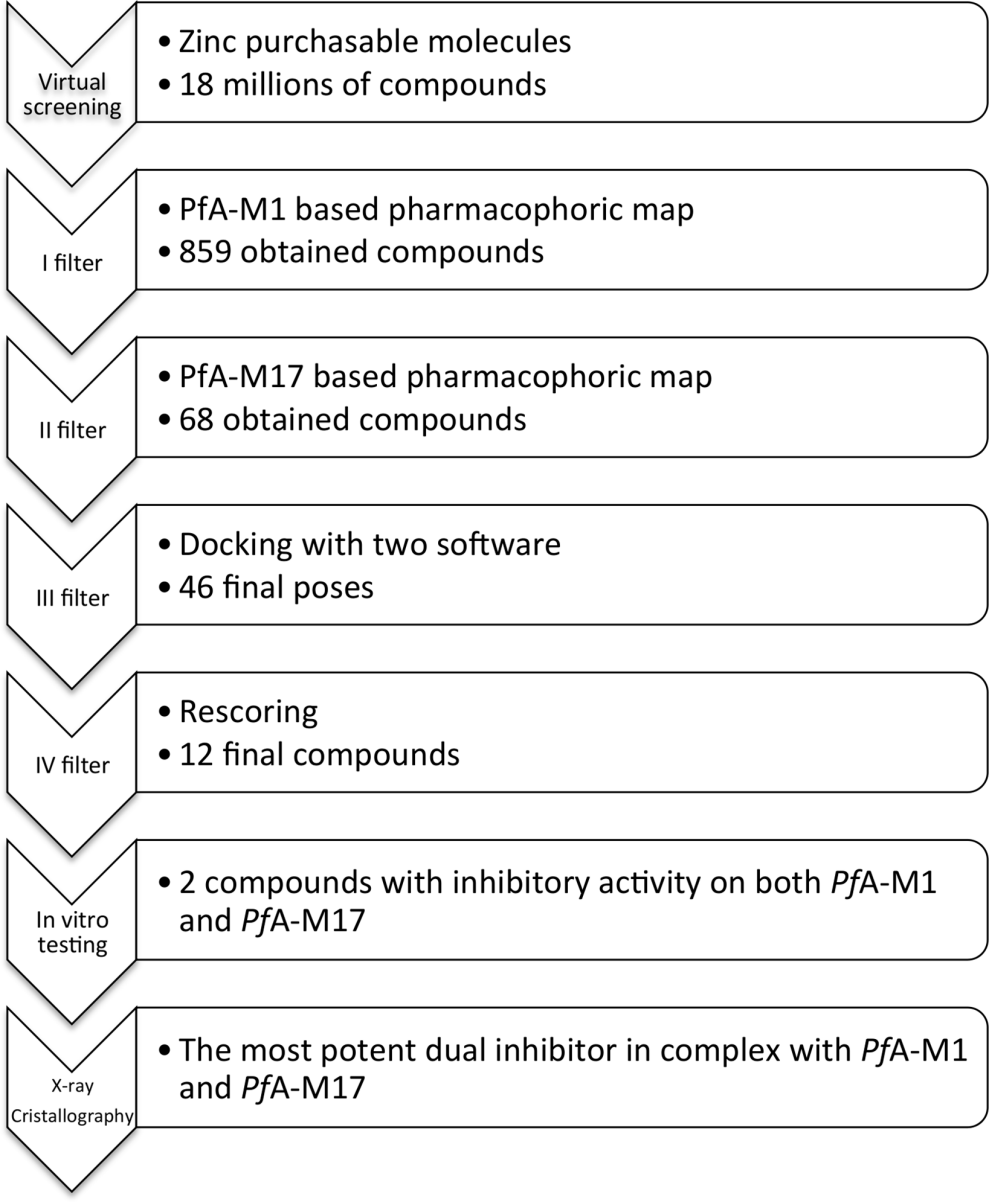
Virtual screening workflow.

The two pharmacophore maps were then used to screen the ~18 million purchasable compounds of the ZINC database [17] using a hierarchical screening protocol (Fig 2). The initial screen of the ZINC database with the *Pf*A-M1 pharmacophore map identified 859 molecules that satisfied the required pharmacophore features. From the results of this screen, a new database of conformations was generated, and subsequently screened against the *Pf*A-M17 pharmacophore map. This filtered screen identified 68 molecules that satisfied our requirements for both pharmacophores (Table A in S1 File). The obtained virtual hits were each visually inspected to check the presence of a known zinc-binding group (ZBG), as would be expected for a potential *Pf*A-M1/*Pf*A-M17 inhibitor.

### Molecular docking

The 68 molecules that satisfied the critical features of *Pf*A-M1 and *Pf*A-M17 pharmacophores were then investigated further by molecular docking. Compounds were filtered using two distinct docking tools, Molegro Virtual Docker (MVD) [21] and FlexX [22]. First the 68 virtual hits were docked to *Pf*A-M1 and *Pf*A-M17 using MVD. On the basis of the Moldock score [21], as well as the ability of these compounds to coordinate the zinc ions of the catalytic sites of *Pf*A-M1 and *Pf*A-M17 (evaluated by visual inspection), 22 molecules were discarded (Table A in S1 File). The remaining 46 molecules were then subjected to a second docking simulation using FlexX [22]. Docking of metalloproteins still remains a challenge due to the multiple coordination geometries of the zinc ion and the lack of specific force field parameters to model the metal-ligand interactions [30]. In this scenario, FlexX is described as being able to detect and assign the statistically significant docking poses while simultaneously selecting the best metal coordination geometry. All ligand poses resulting from FlexX docking were further inspected in order to determine if they were similar to the MVD poses. Nine poses were rejected because they had a RMSD > 2.0 Å compared to MVD poses. A total of 37 drug-like molecules showed comparable poses when docked with both MVD and FlexX (RMSD < 2.0 Å on superposed heavy atoms) in *Pf*A-M1 and *Pf*A-M17 binding sites. To rank the 37 virtual hits we used the Hyde scoring function [23] to predict the binding affinity of each compound to *Pf*A-M1 and *Pf*A-M17 (Table A in S1 File). This scoring function takes into account the zinc coordination geometry contribution in the estimation of the binding affinity [23]. On the basis of the binding affinity predicted with the Hyde scoring function, and final visual inspection, we selected 12 molecules to investigate *in vitro* (Table B in S1 File).

### Evaluation of the inhibitory activity of selected compounds against *Pf*A-M1 and *Pf*A-M17

**Fig 3:**
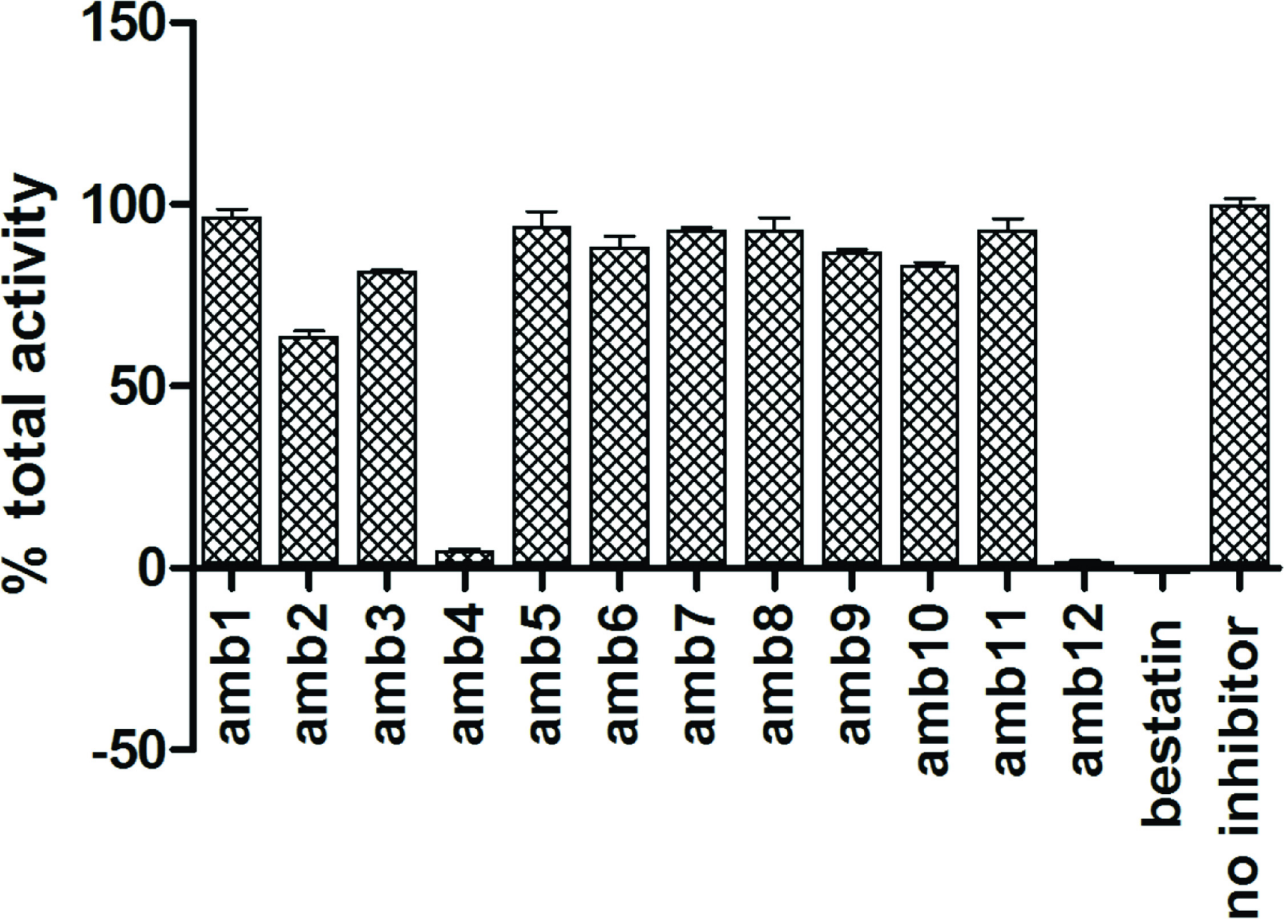
Total aminopeptidase activity in the presence of 12 virtual screen compounds. The graph shown indicates the percentage of total aminopeptidase activity in the presence of 1 mM of compound (as indicated). Bestatin was used as control.

**Fig 4:**
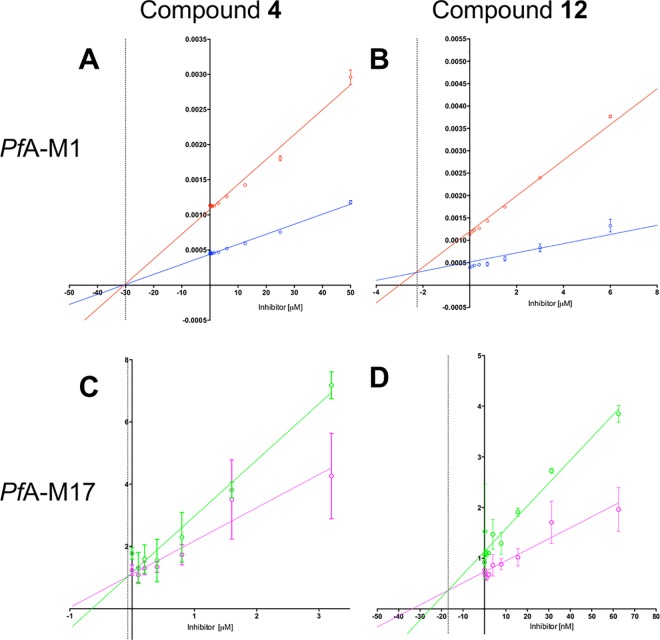
Dixon plots of 1/V (*y axis*) versus inhibitor concentration (*x axis*) for *Pf*A-M1 (top panel) and *Pf*A-M17 (bottom panel). For *Pf*A-M1, substrate concentrations were 20 μM (blue) and 40 μM (red); inhibitor concentration in μM. For *Pf*A-M17, substrate concentrations were 5 μM (magenta) and 10 μM (green); inhibitor concentration in μM or nM as indicated. The point of intersection (-*K*_i_) is indicated as a dotted line. (A) *Pf*A-M1 and compound **4. (B)** *Pf*A-M1 and compound **12. (C)** *Pf*A-M17 and compound **4. (D)** *Pf*A-M17 and compound **12**

The top 12 hits identified in the virtual screen were evaluated for their ability to inhibit both *Pf*A-M1 and *Pf*A-M17 *in vitro*. We initially tested the total aminopeptidase activity (as reported by the fluorigenic substrate L-leucyl-7-amido-4-methylcoumarin) in the presence of 1 mM compound (Fig 3). In this screen, the “no compound” control was considered 100% activity and bestatin was included as an indicator/control for inhibition. From the results obtained, only compounds **4** (ZINC ID: 25108749) and **12** (ZINC ID: 4090432) were able to inhibit the activity of the enzymes to > 50%. Compound **4** was capable of 95% inhibition at 1 mM, while **12** showed 98% inhibition at 1 mM (Fig 3). The two inhibitory compounds were further analyzed to identify their effects on the individual aminopeptidases. To do this, we performed dose-response assays for each inhibitor with each enzyme, and then determined a *K*_i_ via Dixon plots (Fig 4). The most potent inhibitor was compound **12**, with a *K*_i_ of 2.3 μM for *Pf*A-M1 and 17.0 nM for *Pf*A-M17. Compound **4** was a weaker inhibitor of both enzymes with *K*_i_’s in the micromolar range (*K*_i_ = 30.0 μM for *Pf*A-M1 and 0.7 μM for *Pf*A-M17; Fig 4). Both compounds showed a competitive inhibition profile for each enzyme.

### Docking analysis of compounds 4 and 12

**Fig 5:**
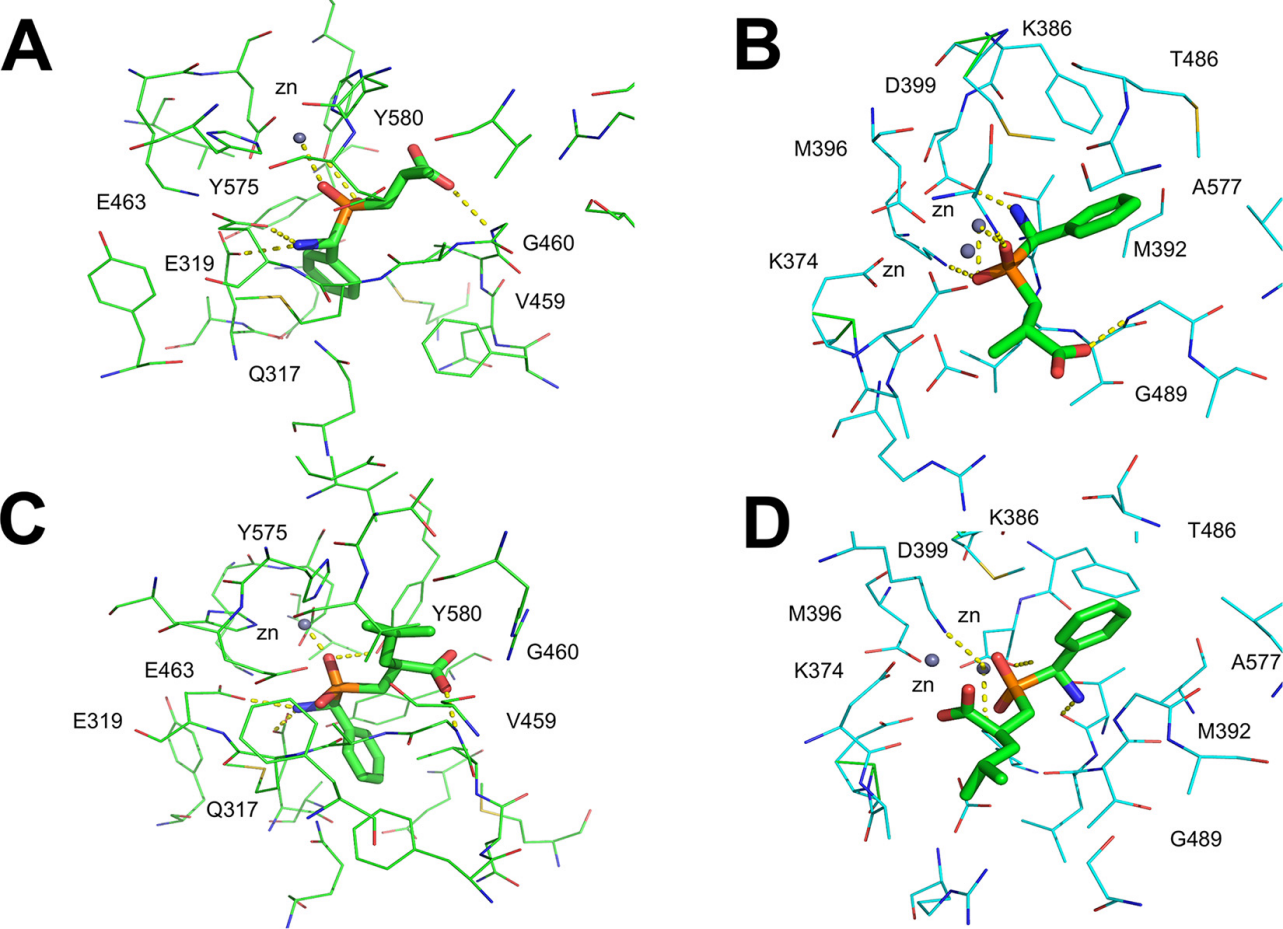
Molecular docking of compounds 4 and 12 shows the predicted binding mode. Compound **4** is shown bound to *Pf*A-M1 (A) and *Pf*A-M17 (B); compound **12** is shown bound to *Pf*A-M1 (C) and *Pf*A-M17 (D). Residue names are indicated.

Compounds **4** (3-{[amino(phenyl)methyl](hydroxy)phosphoryl}-methyl propanoic acid) and **12** (2-({[amino(phenyl)methyl]hydroxy)phosphoryl}methyl)4-methylpentanoic acid) are both organophosphorus acid compounds, well known to be effective metal chelating agents [13]. The binding mode of each compound was predicted by computational docking (Fig 5). The orientation of the poses obtained with compound **4** in the active site of *Pf*A-M1 and *Pf*A-M17 from MVD are comparable with those of FlexX (RMSD between compound **4** poses in *Pf*A-M1: 0.8 Å; RMSD between compound **4** pose in *Pf*A-M17: 0.9 Å). The best poses of compound **4** in *Pf*A-M1 and *Pf*A-M17 active sites are depicted in panel A and B of Fig 5. In the *PfA*-M1 active site, compound **4** was predicted to form the following interactions: a phosphinic oxygen atom coordinates the zinc and is hydrogen-bonded to the side chain of Tyr580; the amino group forms interactions with Glu319 and Glu463. The aromatic ring forms favorable hydrophobic interactions with Tyr575, Gln317 and Val459. The methyl group is accommodated in the hydrophobic cleft formed by Tyr580 and Gly460. Additionally, a hydrogen bond is formed between the oxygen of the carboxylic group and Gly460. The coordination geometry of the zinc ion predicted by FlexX is a trigonal bypiramid.

The molecular docking of compound **4** in *Pf*A-M17 predicts that: (1) the negatively charged phosphinic oxygen coordinates one of the zinc ions and interacts with Lys374 and Lys386, (2) the amino group forms hydrogen bonds with Thr486 and Asp399, and (3) a additional hydrogen bond is formed between Gly489 and the phospinic oxygen. The pose is further stabilized by hydrophobic interactions between the aromatic ring and Met392, Met396, Leu487 and Ala577.

The best poses of compound **12** in *Pf*A-M1 and *Pf*A-M17 active sites are depicted in panel C and D of Fig 5. Again, a reasonably similar binding mode was observed from the two different docking tools (RMSD for *Pf*A-M1: 1.1 Å; RMSD for *Pf*A-M17: 1.5 Å). Compound **12** in *Pf*A-M1 active site is predicted to coordinate the zinc ion with the negatively charged oxygens of its phosphinic group, which interact also with Tyr580. Glu319 and Glu463 form hydrogen bonds with the amino group and the pose is further stabilized by a hydrogen bond between Gly460 and the oxygen of the carboxylic group. Hydrophobic interactions are observed between Gly317, Tyr575, Val459, Ala461 and the aromatic ring, and additionally between the methyl group and Glu497. The compound is extended into the hydrophobic cleft, filling the S1 subsite of the large active site cavity.

Compound **12** was docked into the *Pf*A-M17 active site with its the aromatic ring in the hydrophobic cleft formed by residues Met392, Met396, Phe398, Ala577, Gly489. The two zinc ions of *Pf*A-M17 are coordinated by one of the oxygens of the phosphinic moiety and by one of the oxygen atoms of the carboxylic group of compound **12**, while the other oxygen of the carboxylic group is hydrogen-bonded to Lys386. The amino group of compound **12** forms a hydrogen bond with Thr486. The aliphatic hydrophobic tail is extended in the apolar cleft, filling the S1 subsite of the active site cavity.

### Crystal structures of *Pf*A-M1 and *Pf*A-M17 in complex with compound 12

**Fig 6:**
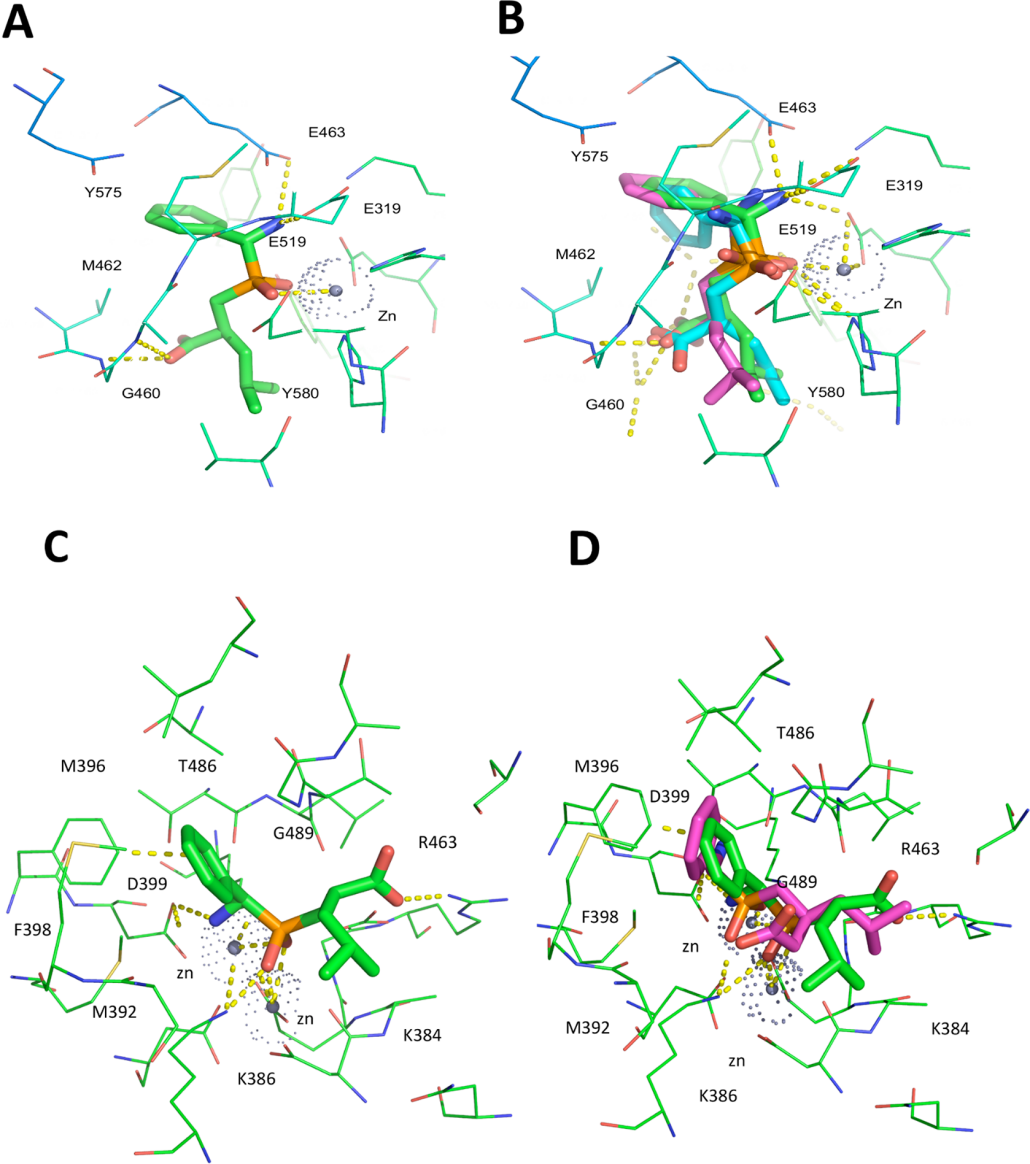
X ray crystal structures of compound 12 bound to *Pf*A-M1 and *Pf*A-M17. (**A**) View from the top of the catalytic pocket, showing compound **12** and the main interactions in the *Pf*A-M1 active site (in green). (**B**) Structural alignment of compound **12** as experimentally determined (green), FlexX-based pose (in blue) and MVD-based pose (in pink). (**C**) View from the top of the catalytic pocket, showing compound **12** (green) and the main interactions in the *Pf*A-M17 active site. (**D**) Structural alignment of compound **12** as experimentally determined (green), and the most similar predicted pose (pink).

The X-ray crystal structures of the potent compound **12** in complex with both *Pf*A-M1 and *Pf*A-M17 were solved to a resolution of 2.0 and 2.3 Å respectively (Table 1). Compared to unliganded *Pf*A-M1 (PDB ID: 3EBG), no gross rearrangement was observed upon binding of compound **12**. Examination of *Pf*A-M1 in complex with compound **12** revealed the molecular basis for the inhibitory activity of this compound. The inhibitor coordinates zinc through the oxygen atoms of the phosphinate group (Fig 6A). The oxygen of the central phosphinate moiety also forms hydrogen bond interactions with Tyr580, and the amino group forms hydrogen bonds with Glu319, Glu519 and Glu463. Furthermore, a hydrogen bond is formed between Gly460 and the oxygen of the carboxylic group. The aromatic ring forms favorable hydrophobic contacts with Val459, Met462 and Tyr575. In total, the compound forms five hydrogen bonds and is further stabilized by hydrophobic interactions.

Superposition of the compound **12** crystallographically determined binding pose with those predicted by FlexX and MVD, show strong agreement between the computationally predicted binding modes and the crystallographic data (Fig 6B). The zinc-binding group is in the same position in all the three cases and there is only a small difference in the position of the aromatic ring, which is rotated in the pose predicted by MVD. The highest correspondence between docking pose and crystal structure was obtained with FlexX software.

In *Pf*A-M17, compound **12** binds with a different chirality (*R*) at its C11 atom, compared to the topologically equivalent atom ((*S)-*C19) of its structural analogue hPheP[CH_2_]Phe, bound to *Pf*A-M17 (PDB Code: 3KR5) (Fig 6C). With such chirality, compound **12** is still able to orient its amino moiety towards Asp399 (interacting with the latter *via* a hydrogen bond) and at the same time to direct the phenyl substituent towards the hydrophobic pocket formed by Met392, Met396, Phe398, Gly489, Leu492 and Ala577. As in the case of hPheP[CH_2_]Phe, both zinc ions of *Pf*A-M17 are tightly coordinated to one or both of the phosphinate oxygens of compound **12,** and the latter can also form a hydrogen bond to the catalytic carbonate. Disordered electron density for the carboxylate and leucyl substituents of compound **12** is observed in all twelve chains of the *Pf*A-M17 binding pockets, indicating substantial flexibility of binding. As a result, the complete compound could be build in only one of the active sites (Chain E), and even then could only be modeled with half occupancy for the flexible carboxylate and leucyl groups. In this pose, the carboxylate forms a hydrogen bond with the main chain amine of Gly489, while the aliphatic leucyl group makes no interactions and is exposed to solvent.

In contrast to the structure of *Pf*A-M1 bound to compound **12**, there is some discrepancy between the docked and crystallographically determined binding poses of compound **12** bound to *Pf*A-M17 (Fig 6D). Unlike the experimentally determined structure, the docked pose shows that only a single zinc is coordinated by the phosphinic moiety. The amine moiety is also oriented differently: rather than forming a hydrogen bond with Asp399, it is directed in the opposite direction and forms hydrogen bonds to the main chain oxygen atoms of Thr486 and Leu487. Irrespective of the different zinc coordination, the phenyl substituent of compound **12** is bound in largely the same position in the docked structure compared to the crystal structure, sandwiched between Met396 and the main chain of Gly489. Finally, the docked structure showed few interactions between the leucyl tail of compound **12** and the binding pocket. This lack of binding interactions is also observed in the crystal structure, and accounts for the flexibility of this compound region. Composite OMIT maps of compound **12** when bound to M1 and M17 (S1 Fig) were consistent with our previous observations and showed discorded electron density of the Leu sidechain when in complex with both M1 and M17. This indicates that, similarly to the compound **12**-M17 binding conformation, there is flexibility of binding also in compound **12**-M1.

## Discussion

Malaria is currently one of the most deadly infectious human diseases [1]. Emerging resistance to front-line antimalarials, including the artimisinins, presents an urgent need for new antimalarial drugs. *Pf*A-M1 and *Pf*A-M17 are essential for parasite survival and are validated antimalarial drug targets [7–10]. To date, different inhibitor scaffolds have been described as competitive dual inhibitors of *Pf*A-M1 and *Pf*A-M17, including peptide-based bestatin analogues, phosphinopeptides and phosphonic acids [7–9, 11–14]. Recently, our group has also identified hydroxamic acid containing compounds as potent inhibitors of *Pf*A-M1 and *Pf*A-M17 that show anti-parasitic activity in the nanomolar range [12].

In the present study, we explored the ability of a new structure-based virtual screening protocol to identify dual inhibitors of *Pf*A-M1 and *Pf*A-M17. Virtual screening is highly desirable for structure-based drug design (SBDD) in that it potentially has the ability to rapidly accelerate and economize drug discovery. Computational approaches can identify potential hits in a fraction of the time and effort that is required for high-throughput *in vitro* screening approaches. However, despite a number of successful SBDD studies that have incorporated *in silico* approaches [31,32], computational early lead discovery still suffers from several limitations [33, 34]. This is largely a result of *in silico* results not being experimentally validated and therefore methodologies and approaches are not evolving as is required. The ultimate proof of concept required for molecular docking and virtual ligand screening is represented by the experimentally determined structure of the complex between the target and virtual hits, which is rarely determined and published [31, 32].

The main goal of our current work, therefore, is twofold, i) the identification of novel dual inhibitors of *Pf*A-M1 and *Pf*A-M17 and ii) the experimental validation of the applied structure-based virtual screening protocol. Starting from the available structural data, two pharmacophore hypotheses have been developed, and used to screen the ZINC database. Subsequently, a docking simulation has been carried out using two different docking tools, and several filters have been applied to finally select promising hits. We identified twelve compounds that satisfied all the filtering criteria. Interestingly, some of them contain chemical scaffolds already associated with other metalloaminopeptidase inhibitors, providing a further validation of the computational results. Two of the identified molecules demonstrated inhibitory activity for both *Pf*A-M1 and *Pf*A-M17. In particular, compound **12** acted as a low nanomolar *Pf*A-M17 inhibitor (*K*_i_ = 17.0 nM). The comparison of crystal structure of the phosphonic arginine mimetics compounds series [13] recently identified by our group with the inhibitors identified herein shows a similar pattern of interactions with the zinc ion, involving the oxygen atoms of the phosphonic/phosphinic moiety. Also, a hydrogen bond with Tyr580 and the O1 atom of the phosphinic/phosphinic group is conserved. The most potent inhibitor of phosphinic arginine derivatives series showed a *K*_i_ = 104 uM for *Pf*A-M1 and *K*_i_ = 11 nM for *Pf*A-M17. The higher potency of compound **12** as a *Pf*A-M1 inhibitor (*K*_i_ = 2.3 uM) could potentially be explained by the entropy gain of binding due to the lack of a flexible linker between the aromatic moiety and the aminophosphinic moiety.

The crystal structure of *Pf*A-M1 in complex with compound **12** further confirmed the validity of the computational screening described herein. In contrast to the structure of *Pf*A-M1 bound to compound **12**, we noticed some discrepancy between the docked and structurally determined binding poses of compound **12** bound to *Pf*A-M17. Investigating the reasons underlying the disagreement between the docked and structurally determined binding poses of compound **12** in complex with *Pf*A-M17, we found that the original compound retrieved from the ZINC Database (ZINC ID: 04090433) during the virtual screening process showed, at its C11 atom, the same chirality (*S*) of its structural analogue hPheP[CH_2_]Phe. This is not surprising considering that the latter was used as a template to build the 3D-pharmacohopre map used during the screening. The chirality difference between the docked compound and the experimentally determined binding pose is, therefore, probably responsible for the observed discrepancy. Indeed, as a consequence of the incorrect predicted chirality, the amino moiety of ZINC04090433 cannot be oriented properly without causing the adjacent phenyl substituent of the C11 atom to severely clash the active site residues Phe398 and Thr486. Moreover, with the wrong C11 chirality and the phenyl moiety accommodated in the hydrophobic cleft formed by residues Met392, Met396, Phe398, Gly489, Leu492 and Ala577, the phosphinate moiety of ZINC04090433 results shifted of ∼ 1.5Å from the experimentally determined position, thereby losing a coordination site with the Zn ions of *Pf*A-M17, and leaving the room for the carboxylic group to coordinate the other Zn ion.

We are unable at the moment to provide a clear explanation to the inability of the 3D-pharmacophore map to retrieve the right enantiomer (*R*) of compound **12** from the ZINC Database (ZINC12888856). Nonetheless, a retrospective analysis of the docking results, in light of the experimentally determined complex between M17 and compound (*R*)**12**, showed that a low scoring pose of the (*S*) enantiomer of compound **12** (ZINC04090433) (S2 Fig) docked with a conformation highly similar to (*R*)**12**, as experimentally determined. In this case however, the aromatic moiety at C11 position of compound (*S*)**12** is predicted to be too close to the aromatic side chain of Phe398 (~3.0 Å), compared to the crystal structure (~3.5 Å) of (*R*)**12**. The latter observation accounts for the low energy score observed for this pose.

Despite the overall success of our protocol, we did not identify a completely new chemical scaffold or ZBG, as both our hit compounds coordinates the zinc ion through their phosphinate group, as previously described [13]. One of the possible reasons of this failure can be related to the excessively stringent criteria imposed by the two pharmacophore maps. The latter indeed may bias the results to compounds highly similar to the initial ones used to derive the maps. Further, the presence of the metal ions in the active sites still represents a great challenge for currently available docking tools. For many docking scoring functions, which mostly rely on non-bonded interactions, the partial covalent nature of metal-ligand interactions remains problematic [30].

Despite these limits, the experimental validation of our results supports our computational methodology. Compounds **4** and **12** were the top-ranking hits according to MVD and Hyde scores. Moreover, there is a strong agreement between the predicted binding mode of compound **12** to *Pf*A-M1 and the crystallographic data.

## Conclusion

Our described virtual screening protocol identified a potent dual inhibitor of the *Pf*A-M1 and *Pf*A-M17 proteins. These results indicate that the virtual screening protocols can be successfully applied in other studies, and the obtained structure can be used as starting point for further development and optimization of new lead molecules.

## Supporting Information

S1 Fig Simple composite omit map of (A) *Pf*A-M1-12 active site contoured to 0.7 σ, and (B) *Pf*A-M17-12 active site contoured to 0.9 σ. (PDF)

S2 Fig Structural alignment of compound 12 as experimentally determined (green), and the most similar predicted pose (violet) in *Pf*A-M17 active site. (TIF)

S1 File Compounds retrieved through virtual screening (Table A). Compounds retrieved throughout screening of *Pf*A-M1 and *Pf*A-M17 (Table B).
(DOCX)
