# Supplementary figures and images for: Identification and Validation of a Potent Dual Inhibitor of the P. falciparum M1 and M17 Aminopeptidases Using Virtual Screening
Source: PLoS One. 2015 Sep 25;10(9):e0138957. doi: 10.1371/journal.pone.0138957 (PMC4583420; doi:10.1371/journal.pone.0138957)

**A**

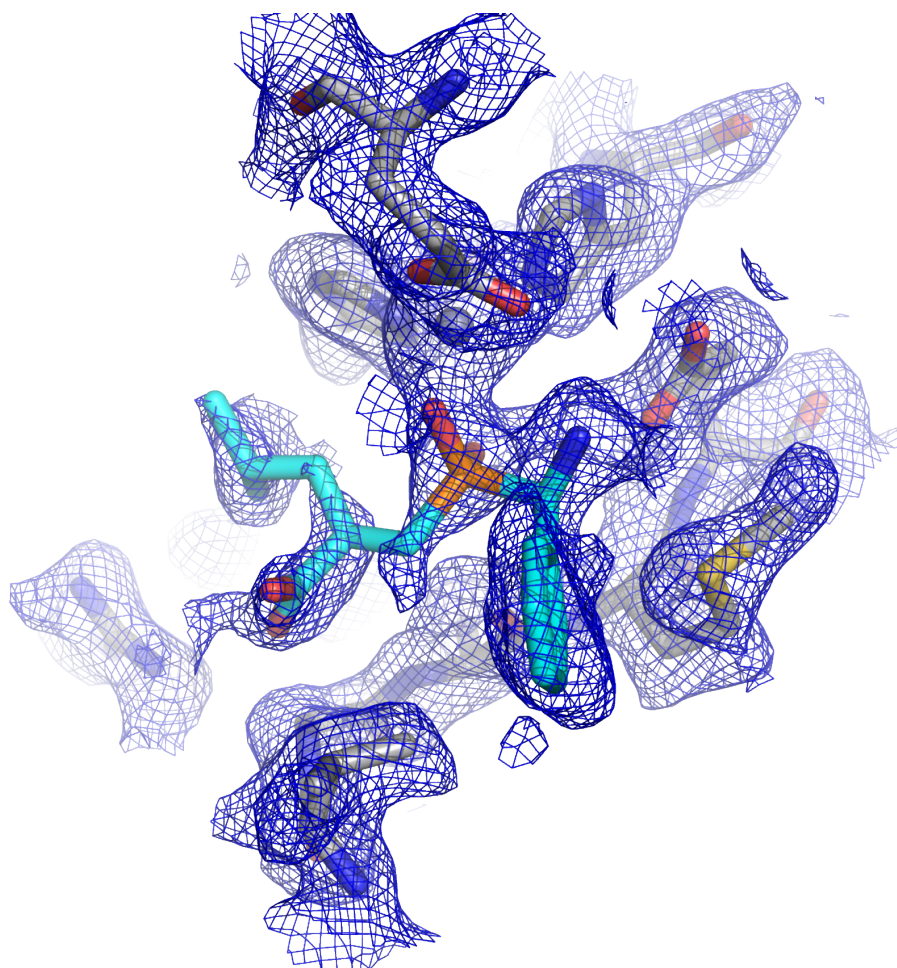

**B**

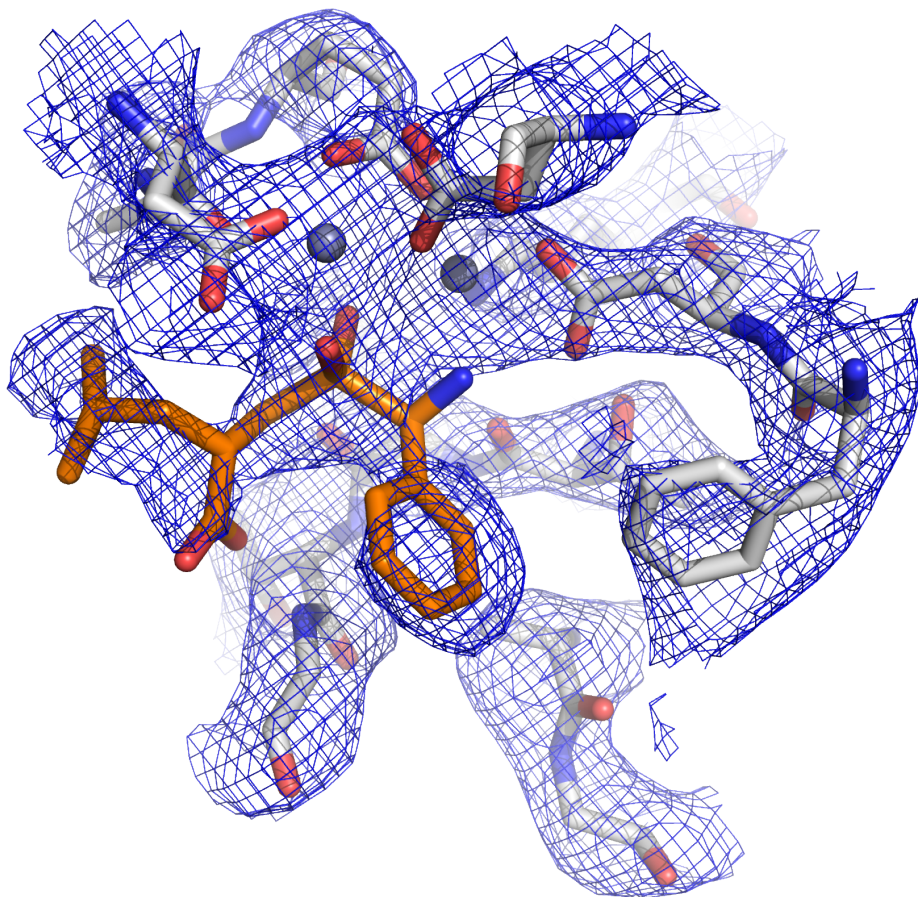

Supplement: S1 Fig — (PDF) [file pone.0138957.s001.pdf]

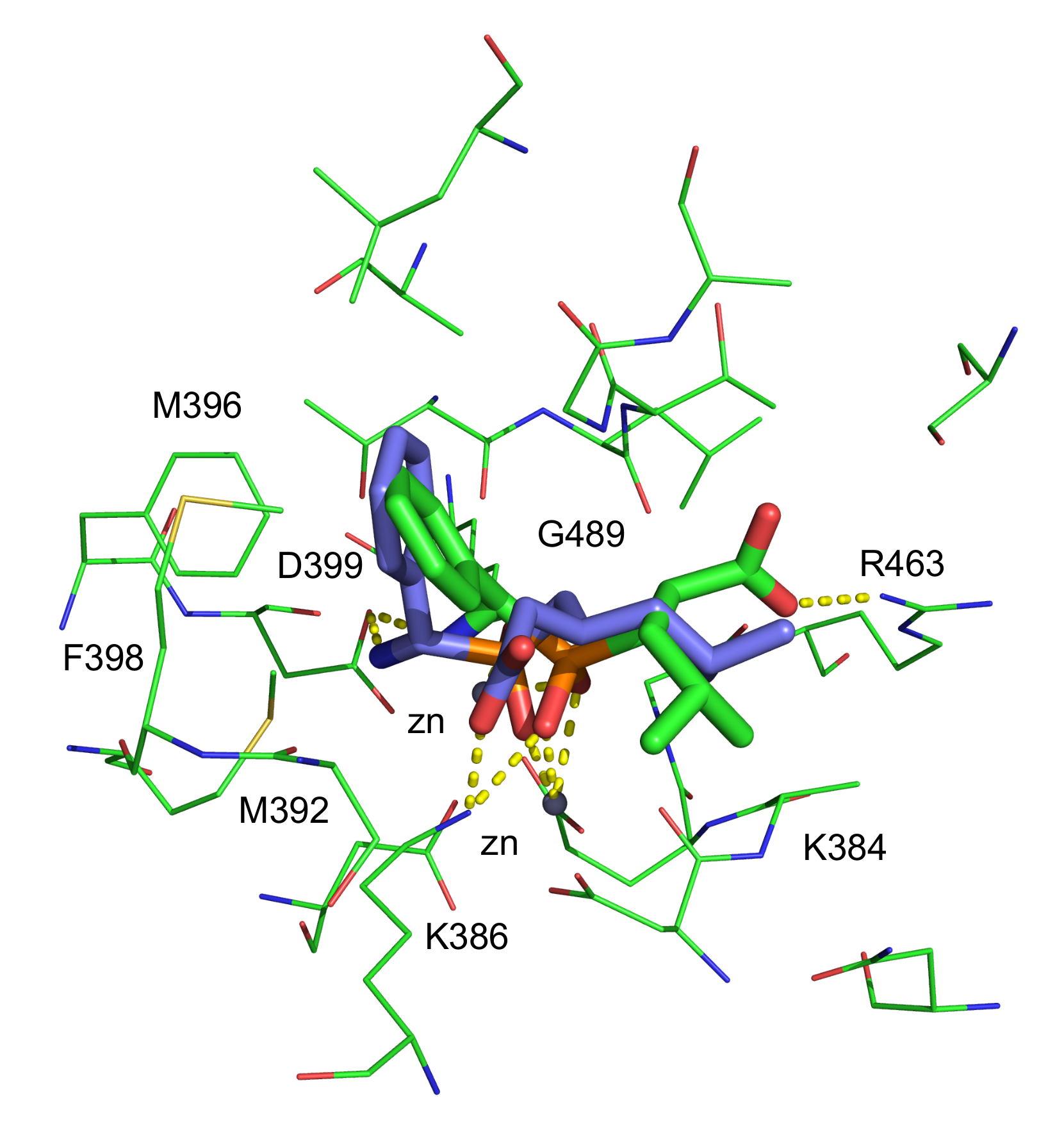

Supplement: S2 Fig — (TIF) [file pone.0138957.s002.tif]
