# Supplementary material for: Identification and Validation of a Potent Dual Inhibitor of the P. falciparum M1 and M17 Aminopeptidases Using Virtual Screening
Source: PLoS One. 2015 Sep 25;10(9):e0138957. doi: 10.1371/journal.pone.0138957 (PMC4583420; doi:10.1371/journal.pone.0138957)
Supplement: S1 File — Compounds retrieved throughout screening of PfA-M1 and PfA-M17 (Table B). (DOCX) [file pone.0138957.s003.docx]

**Table A.** Compounds retrieved through virtual screening.

| **Compound ID** | **PfA-M17** | | | **PfA-M1** | | |
| --- | --- | --- | --- | --- | --- | --- |
|  | **MVD Score** | **FlexX**  **Score** | **Hyde Score** | **MVD Score** | **FlexX**  **Score** | **Hyde Score** |
|  |  |  |  |  |  |  |
| **ZINC01709622** | **-101.90** | **-41.60** | **-35.00** | **-116.75** | **-31.63** | **-56.00** |
| ZINC59382697 | -102.61 | -44.70 | -12.00 | -111.29 | -21.06 | 2.00 |
| **ZINC04858049** | **-106.62** | **-35.73** | **-37.00** | **-104.50** | **-65.82** | **-33.00** |
| ZINC19817652 | -92.74 | n.d. | n.d. | -104.15 | n.d. | n.d. |
| **ZINC71791152** | **-113.59** | **-24.59** | **-22.00** | **-103.50** | **-24.59** | **-19.00** |
| ZINC20264671 | -102.66 | -40.97 | 0.00 | -103.33 | -24.89 | 0.00 |
| ZINC20264673 | -99.31 | -35.40 | -7.00 | -103.17 | -25.02 | 0.00 |
| **ZINC20756702** | **-102.62** | **-27.87** | **-32.00** | **-101.20** | **-25.70** | **-35.00** |
| ZINC20264675 | -90.91 | -37.62 | -8.00 | -100.60 | -27.54 | -4.00 |
| **ZINC20112688** | **-97.07** | **-31.21** | **-23.00** | **-100.34** | **-29.88** | **-29.00** |
| ZINC20264676 | -95.83 | -34.35 | -26.00 | -100.34 | -26.23 | 14.00 |
| ZINC20610912 | -87.60 | -25.29 | -15.00 | -99.10 | -25.10 | 7.00 |
| ZINC20610914 | -77.74 | n.d. | n.d. | -99.02 | n.d. | n.d. |
| ZINC20756706 | -85.58 | -46.89 | -15.00 | -98.95 | -40.24 | -11.00 |
| ZINC08700676 | -77.05 | n.d. | n.d. | -97.88 | n.d. | n.d. |
| ZINC20756697 | -97.76 | -43.29 | -15.00 | -97.73 | -36.71 | -23.00 |
| ZINC20756712 | -98.43 | -41.05 | -2.00 | -96.35 | -35.50 | -6.00 |
| **ZINC25108749** | **-100.99** | **-35.46** | **-48.00** | **-95.75** | **-32.14** | **-40.00** |
| ZINC25108737 | -84.42 | n.d. | n.d. | -95.73 | -42.99 | -15.00 |
| ZINC25108846 | -109.10 | -34.74 | -27.00 | -94.77 | -35.38 | -20.00 |
| ZINC04580672 | -87.21 | -36.41 | -9.00 | -94.16 | -34.64 | -5.00 |
| **ZINC20757425** | **-100.89** | **-26.76** | **-21.00** | **-92.21** | **-31.88** | **-20.00** |
| **ZINC20112455** | **-107.78** | **-36.89** | **-26.00** | **-92.01** | **-32.45** | **-41.00** |
| ZINC20112501 | -74.00 | n.d. | n.d. | -91.70 | n.d. | n.d. |
| ZINC01631050 | -79.97 | n.d. | n.d. | -91.51 | n.d. | n.d. |
| ZINC19817655 | -104.10 | -39.97 | -26.00 | -91.38 | -30.10 | -23.00 |
| ZINC34650940 | -79.92 | n.d. | n.d. | -89.54 | n.d. | n.d. |
| ZINC20757419 | -87.60 | -28.63 | -16.00 | -89.55 | -35.90 | -14.00 |
| ZINC19995385 | -99.64 | -25.61 | -16.00 | -89.770 | -21.28 | -34.00 |
| ZINC01731778 | -89.73 | n.d. | n.d. | -88.63 | n.d. | n.d. |
| ZINC38200731 | -84.00 | -32.27 | -15.00 | -87.84 | -30.27 | -2.00 |
| **ZINC00266007** | **-100.34** | **-27.44** | **-25.00** | **-83.81** | **-22.51** | **-20.00** |
| ZINC13539556 | -82.99 | -23.78 | -17.00 | -83.59 | -32.40 | 2.00 |
| ZINC12650469 | -81.90 | n.d. | n.d. | -83.54 | n.d. | n.d- |
| ZINC19300333 | -91.06 | -28.17 | -25.00 | -82.27 | -24.91 | -24.00 |
| ZINC20610916 | -88.22 | -30.07 | -9.00 | -82.24 | -26.01 | -3.00 |
| ZINC02044813 | -76.18 | n.d. | n.d. | -82.20 | n.d. | n.d. |
| ZINC19300334 | -92.69 | -27.72 | -19.00 | -82.17 | -21.78 | -19.00 |
| ZINC20827879 | -91.86 | -25.91 | -17.00 | -81.92 | -20.33 | -23.00 |
| ZINC00895124 | -81.66 | n.d. | n.d. | -81.58 | n.d. | n.d. |
| ZINC01529598 | -85.49 | n.d. | n.d. | -81.04 | n.d. | n.d. |
| **ZINC71783874** | **-107.35** | **-49.73** | **-32.00** | **-80.69** | **-29.89** | **-48.00** |
| ZINC19632644 | -105.29 | -32.03 | -31.00 | -80.37 | -26.90 | -17.00 |
| ZINC39306146 | -87.44 | -30.13 | -27.00 | -80.30 | -28.05 | 20.00 |
| ZINC72187796 | -84.08 | -34.14 | -18.00 | -80.29 | -28.05 | 0.00 |
| ZINC37632378 | -94.97 | n.d. | n.d. | -79.37 | n.d. | n.d. |
| ZINC19805027 | -82.49 | -32.18 | -19.00 | -78.53 | -27.26 | 5.00 |
| ZINC20827883 | -85.92 | -31.40 | -40.00 | -77.81 | -21.66 | -13.00 |
| ZINC75154381 | -92.38 | -36.24 | -11.00 | -76.99 | -29.58 | 9.00 |
| ZINC38610641 | -117.09 | -39.46 | -26.00 | -76.93 | -44.07 | -7.00 |
| ZINC01656225 | -96.09 | -32.32 | -19.00 | -76.84 | -26.87 | -7.00 |
| ZINC19805024 | -92.39 | -29.71 | -29.00 | -76.27 | -28.82 | 1.00 |
| ZINC02193449 | -64.75 | n.d. | n.d. | -74.27 | n.d. | n.d. |
| ZINC66339601 | -87.00 | -30.86 | -20.00 | -74.27 | -25.78 | 4.00 |
| ZINC22055357 | -76.72 | n.d. | n.d. | -74.06 | n.d. | n.d. |
| ZINC23484990 | -80.55 | n.d | n.d | -73.32 | n.d | n.d. |
| ZINC20534262 | -79.39 | n.d. | n.d. | -72.33 | n.d. | n.d. |
| **ZINC33975269** | **-113.11** | **-32.78** | **-28.00** | **-67.48** | **-32.76** | **-38.00** |
| ZINC03652238 | -78.85 | n.d. | n.d. | -67.46 | n.d. | n.d. |
| ZINC19327428 | -56.38 | n.d. | n.d. | -67.06 | n.d. | n.d. |
| ZINC67173548 | -88.01 | -30.15 | -13.00 | -66.54 | -22.46 | 13.00 |
| **ZINC04580676** | **-112.83** | **-35.54** | **-29.00** | **-65.45** | **-32.84** | **-31.00** |
| ZINC13525354 | -73.53 | n.d. | n.d. | -65.26 | n.d. | n.d. |
| ZINC00896091 | -78.93 | n.d. | n.d. | -64.75 | n.d. | n.d. |
| ZINC13756317 | -89.68 | -32.48 | -14.00 | -59.10 | -22.27 | 10.00 |
| ZINC25108743 | -94.09 | -35.79 | -31.00 | -35.27 | -35.86 | -7.00 |
| ZINC25108731 | -98.94 | -7.00 | -16.00 | -34.61 | -32.27 | -15.00 |
| ZINC12428291 | -71.56 | n.d. | n.d. | -32.36 | n.d. | n.d. |

*In bold = 12 final hits; n.d.= 22 excluded compounds*

**Table B.** Compounds retrieved throughout screening of *Pf*A-M1 and *Pf*A-M17 virtual hits.

| **Hit** | **Amb#** | **Zinc#** |  | ***Pf*A-M1** | | | ***Pf*A-M17** | |
| --- | --- | --- | --- | --- | --- | --- | --- | --- |
|  |  |  |  | **ΔG**  **(kJ/mol)** | | **Ki (μM)** | **ΔG**  **(kJ/mol)** | **Ki (μM)** |
| **1** | 544298 | 0112455 | 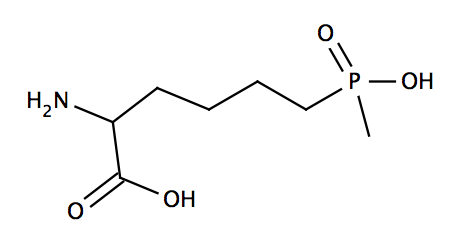 | -26 | n.i. | | -41 | n.i. |
| **2** | 544704 | 20112688 | 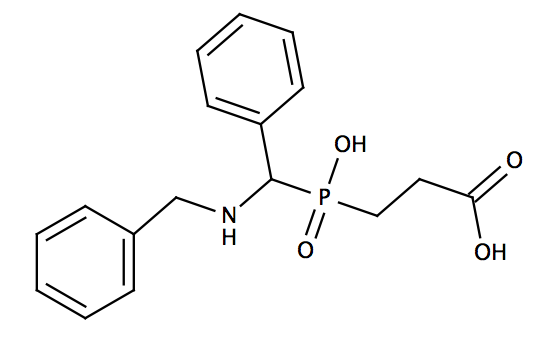 | -23 | n.i. | | -29 | n.i. |
| **3** | 567616 | 20757602 | 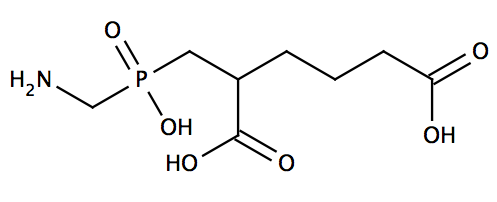 | -32 | n.i. | | -35 | n.i. |
| **4** | 567688 | 25108749 | 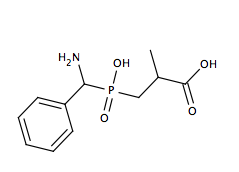 | -48 | 30.0 | | -40 | 0 .7 |
| **5** | 622794 | 01709622 | 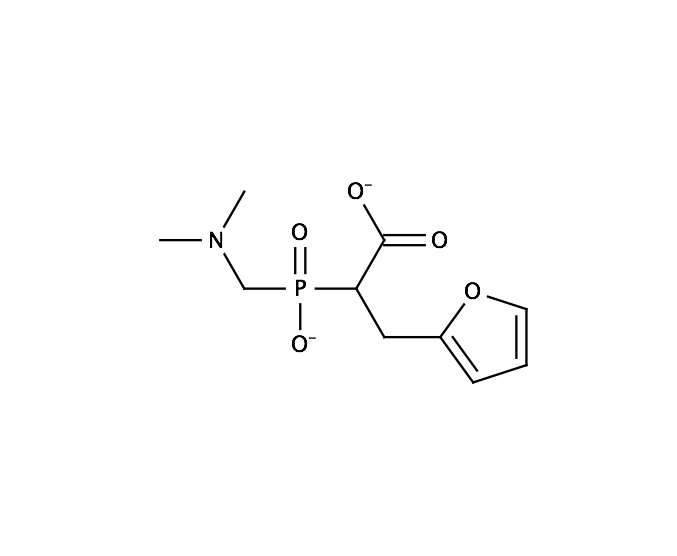 | -35 | n.i. | | -56 | n.i. |
| **6** | 625430 | 20757425 | 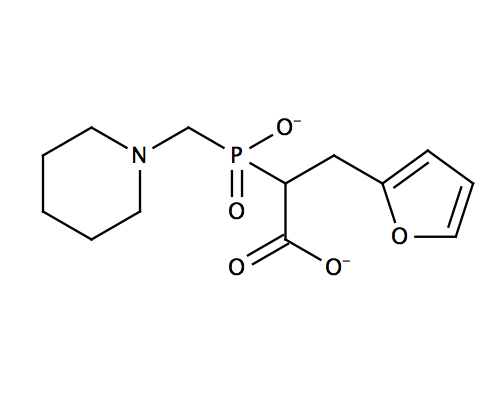 | -21 | n.i. | | -20 | n.i. |
| **7** | 1782095 | 71791152 | 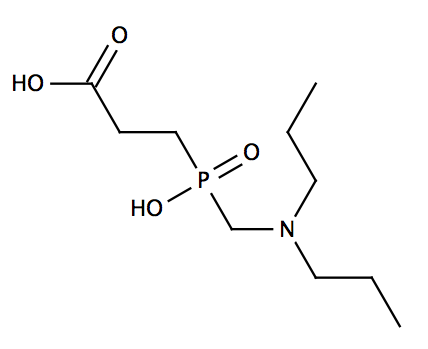 | -22 | n.i. | | -19 | n.i. |
| **8** | 1792132 | 04580676 | 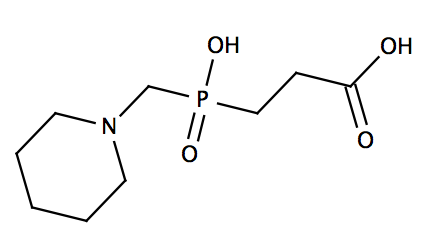 | -29 | n.i. | | -31 | n.i. |
| **9** | 2228993 | 00266007 | 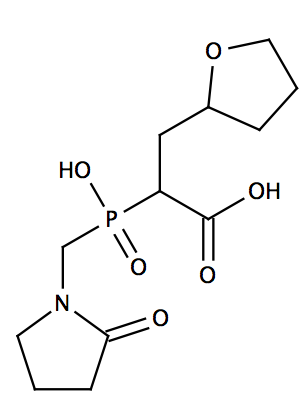 | -25 | n.i. | | -20 | n.i. |
| **10** | 6600046 | 04858049 | 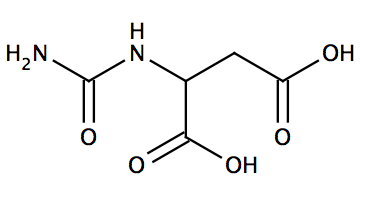 | -37 | n.i. | | -33 | n.i. |
| **11** | 10807462 | 33975269 | 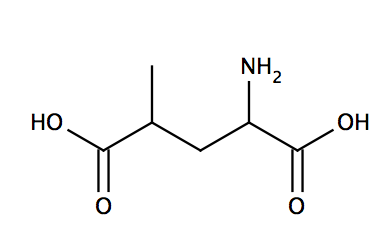 | -28 | n.i. | | -38 | n.i. |
| **12** | 21919335 | 4090433 | 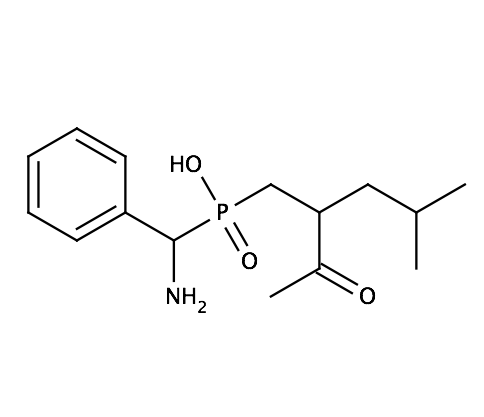 | -32 | 2.3 | | -48 | 0.017 |

**S1 Fig.** Simple composite omit map of (A) *Pf*A-M1-**12** active site contoured to 0.7 σ, and (B) *Pf*A-M17-**12** active site contoured to 0.9 σ.

**S2 Fig.** Structural alignment of compound 12 as experimentally determined (green), and the most similar predicted pose (violet) in *Pf*A-M17 active site.
